# Early or delayed anterior cruciate ligament reconstruction: Is one superior? A systematic review and meta-analysis

**DOI:** 10.1007/s00590-019-02442-2

**Published:** 2019-05-15

**Authors:** D. Ferguson, A. Palmer, S. Khan, U. Oduoza, H. Atkinson

**Affiliations:** 1 Nuffield Department of Orthopaedics Rheumatology and Musculoskeletal Sciences, Botnar Research Centre, Windmill Road, Oxford, OX3 7LD UK; 2 Royal National Orthopaedic Hospital, Stanmore, HA7 4LP UK; 3 Department of Trauma and Orthopaedic Surgery, North Middlesex University Hospital, London, N18 1QX UK

**Keywords:** Anterior cruciate ligament, Reconstruction, Meta-analysis, Timing of reconstruction

## Abstract

**Background:**

Anterior cruciate ligament (ACL) reconstruction is a rapidly developing orthopaedic field and an area of notable clinical equipoise. The optimal timing of surgery in an acute (< 3 weeks) or delayed (≥ 3 weeks) time frame remains unresolved with a 2010 meta-analysis concluding no difference between these two groups across multiple outcomes. In an era of evidence-based medicine, surgeons are still basing their decisions on when to operate on little more than anecdotal evidence and personal preference. Clear guidance is required to determine whether the timing of surgery can optimise outcomes in this largely young and active patient cohort.

**Methods:**

A systematic literature search was performed in January 2018 of Embase, Medline and OpenGrey in accordance with (PRISMA) guidelines. A total of 658 articles were retrieved, with 6 suitable for inclusion, covering 576 ACL reconstructions. Four meta-analyses were performed assessing subjective measures of Tegner activity scale and Lysholm score, and objective measures of arthroscopically identified meniscal and chondral injury. Additional relevant outcome measures underwent narrative review. Study bias was assessed and reported using the Downs and Black checklist.

**Results:**

A statistically significant difference of 0.39 points was found on the Tegner activity scale in favour of early surgery within 3 weeks (RR 0.39, CI 0.10, 0.67, *p *= 0.008). No statistically difference was found between groups for the patient-reported Lysholm score (RR − 0.18, CI − 2.40, 2.05, *p *= 0.17). There was no statistically significant difference between groups for intra-operative findings of meniscal lesions (RR 0.84, CI 0.66, 1.08, *p *= 0.17). A trend towards significance was observed for the incidence of chondral lesions in the early surgery group (RR 0.56, CI 0.31, 1.02, *p *= 0.06). All the studies were rated either fair or good on the Downs and Black checklist with no study excluded due to bias.

**Conclusions:**

Although there was a statistically significant result for the Tegner activity scale in favour of early surgery, the magnitude of the effect is unlikely to translate into any clinically meaningful difference. At present, there remains no clear evidence to determine superiority of acute/early or delayed reconstruction of a ruptured anterior cruciate ligament. Further research through methodologically robust randomised controlled trials or through the UK National Ligament Registry  may help to provide clearer guidance.

## Introduction

The anterior cruciate ligament (ACL) is the most commonly injured knee ligament requiring surgical intervention [1] with estimated national incidences ranging from 8 to 52 cases per 100,000 people per year in the developed world [2–4]. Surgical reconstruction of ruptured ACLs is generally preferred to non-operative management for active individuals, permitting a swift return to function. There has been a rapid evolution in the methods of reconstruction since the inception of ACL surgery in the late 1960s [5]. Present day surgery is technically feasible and successful on a day case basis within 48 h of injury [6] with a median lay-off time of as little as 59.5 days before a return to training, amongst professional athletes [7]. Despite such successes, there remains longstanding controversy and clinical equipoise in a number of key areas relating to an optimal reconstruction; the graft choice of bone patella tendon bone or hamstring autograft [8, 9], single bundle or double bundle grafts [10, 11], the role of biological support in graft maturation [12], and the necessity for bracing during the post-operative rehabilitation, to name a few examples [13, 14],

An additional central tenant in the debate has been timing of intervention [15–17]. Advocates of early intervention in the days to weeks post-injury postulate that a more rapid restoration of tibiofemoral stability in turn reduces further chondral and meniscal damage [18, 19], with a subsequent reduction in degenerative joint disease [20, 21]. This, coupled with the economic advantages of early surgery through a faster return to function [22, 23] are cited as being the key factors influencing superior long-term outcomes. Delayed surgery risks muscle atrophy and deconditioning, thereby potentially slowing rehabilitation. Proponents of delaying intervention for months to years after injury argue that postponing surgery into the inflammation-free period permits a preoperative restoration in range of motion (ROM), ensuring soft tissue optimisation with a resultant reduction in rates of wound complications and arthrofibrosis [15, 24]. An additional advantage of delayed surgery is the ability for patients to prepare psychologically in advance of surgery and establish realistic recovery aims; an important and established factor in successful surgery [15]. The debate around the timing of surgery was recently expanded by a methodologically robust randomised control trial (RCT) questioning the role of surgery in the management of ACL rupture. The authors compared structured rehabilitation plus early ACL reconstruction and rehabilitation, with an optional delayed reconstruction, and found that neither strategy was superior at 2-year or 5-year follow-up, and only 51% of those patients in the delayed reconstruction group actually progressed to surgery [24, 25].

In the most recent systematic review and meta-analysis with the definitions for early and delayed reconstruction set at < 3 weeks and ≥ 6 weeks respectively, the authors found no differences between the groups across a range of patient-reported outcome measures (PROMs) and objective clinical assessments over short to medium-term follow-up [16]. A further more comprehensive systematic review, with much greater study heterogeneity, concluded that there were few or no differences in subjective or objective outcomes between early or delayed groups [17].

### Rationale for performing this review

Since the previous systematic review and meta-analysis in 2010, several studies have been published comparing outcomes of early versus delayed ACL reconstruction [6, 26–30]. The purpose of the present study was to reassess the available literature from the last review to the present day, to determine whether newer evidence is available to support any benefit of early (< 3 weeks) or delayed (≥ 3 weeks) ACL reconstruction in skeletally mature humans. The definitions of early and delayed surgery have been based on the previous systematic review [16] with an amendment in definition for the delayed surgical group to include sub-acute patients in the 3 to 6-week post-injury window; a period where many patients undergo surgery and whose outcomes also warrant inclusion.

Given that early surgery provides faster tibiofemoral stabilisation after injury, reduces joint laxity and the potential for subsequent degenerative changes [18, 19], the alternative hypothesis in our review is that in a population of young healthy adults, early ACL reconstruction is superior to delayed surgery. As per the previous review, the key outcome measures were meniscal/chondral lesions noted at time of reconstruction, PROMs, Lysholm, Tegner, IKDC and VAS scores, and objective clinical examinations of ROM and stability.

## Methods

### Protocol

This systematic review was performed along preferred reporting items for systematic reviews and meta-analyses (PRISMA) guidelines [31].

### Eligibility criteria

**Fig. 1:**
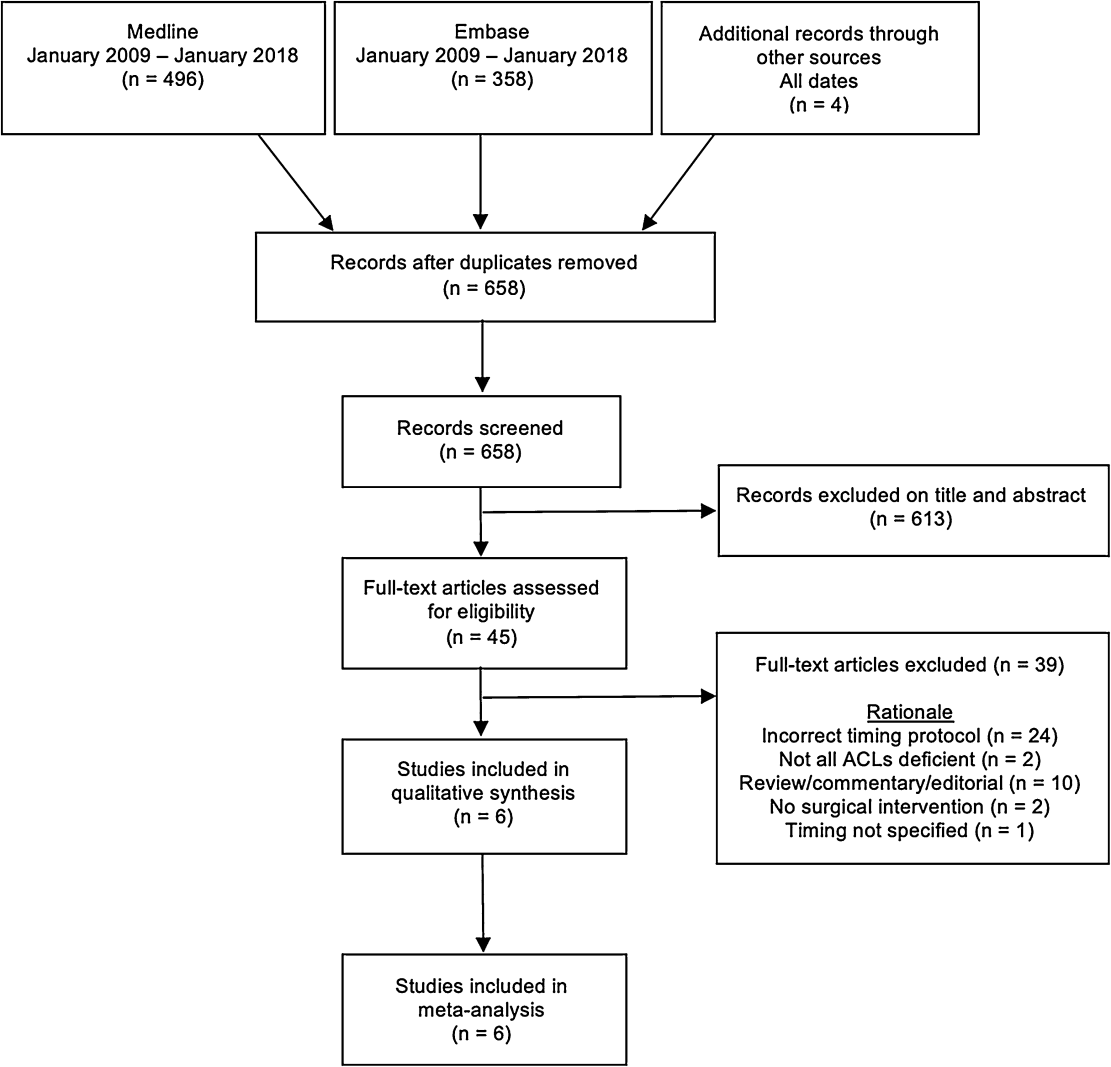
PRISMA flowchart of study selection

Eligibility criteria was specified as English language articles of randomised and non-randomised control trials and comparative cohort studies of ACL-deficient skeletally mature humans published between January 2009 and January 2018. A decision on the timeframe was made to ensure inclusion of all published research since the time of the previous systematic review in 2010. Studies were eligible for inclusion irrespective of open or arthroscopic reconstruction, ACL tear grade, graft type, rehabilitation protocol or gender. Skeletally immature and non-human models were excluded (Fig. 1).

### Search and information source

A scoping search was conducted in early April 2017 with a subsequent decision to expand the retrieval. An electronic database search was conducted in Embase and Medline via the Ovid interface by a senior research librarian at the University of Oxford Cairns Library in May 2017 and updated in January 2018. The strategy consisted of MeSH terms and free text terms (“Appendix 1”). 496 Medline and 358 Embase articles were returned. The only limitation was the year of publication from 2009 to current as this review was subsequent to and built upon the previous systematic review and meta-analysis of Smith 2010 [16]. The following inclusion criteria were applied to all retrieved articles after the removal of duplications: (1) all studies were required to have patients undergoing isolated ACL reconstruction or in combination with a secondary procedure, e.g., meniscectomy; (2) a minimum of one early (surgery < 3 weeks) and one delayed (surgery ≥ 3 weeks) group per study; (3) a minimum of one reported subjective or objective outcome post-intervention; (4) a minimum period of 6-month follow-up. Of the 658 articles, 6 were selected for cross-referencing in PubMed and the Web of Science to identify related articles and explore citation histories. No new articles were identified. A further search on OpenGrey was conducted with no time limit ("Appendix 1"). Review paper reference lists were scrutinised for any further publications not identified in the electronic search and when necessary corresponding authors were contacted for clarification of published work. Editorials, comments, guidelines, case reports, review papers and articles on ACL repair were reviewed but excluded from the analysis. One investigator selected articles meeting inclusion criteria extracting data onto a standardised proforma, and was not blinded to journal names, author names or source.

### Risk of bias assessment

The selected studies comprised a range of randomised control trials, non-randomised trials and observational studies with outcomes across varying time frames ("Appendix 2"). The Downs and Black checklist [32] has previously demonstrated reliability and validity in the assessment of a variety of study designs including observational studies and non-randomised intervention trials [33]. The checklist attributes significant weighting to study design, methodology and statistical power. A modified version of the original checklist was employed with a maximum score of 32. Assessment and scoring was performed by one investigator. The purpose of this was to provide a descriptive summary of sources of bias within the included studies with none excluded on the basis of this assessment.

### Statistical analysis

Four meta-analyses were performed using RevMan V.5.3.5 (The Cochrane Collaboration, Copenhagen, Denmark): for Lysholm score and for Tegner activity scale at final follow-up, and for intra-operative incidence of meniscal and chondral lesions. Continuous variables were extracted and analysed as a mean ± SD. When SD was not reported, the corresponding author was contacted and asked to provide the value. In the case of no response, the study was excluded from the meta-analysis. The mean difference and 95% CI were calculated for continuous variables. Relative risk (RR) and 95% CI were calculated for dichotomous variables. Heterogeneity was measured with *X*^2^ and *I*^2^ statistical tests. Data were pooled using a random-effects model if statistical heterogeneity was ≥ 10% (*I*^2^ test); a fixed-effects model was used if heterogeneity was below 10%. A probability of *p *< 0.05 was deemed statistically significant.

## Results

The search returned 658 citations after the removal of duplications; six studies were assessed as suitable for inclusion in accordance with predefined eligibility criteria. Twenty-four studies included time to surgical intervention in the early group as ≥ 3 weeks thus were excluded. Ten commentaries/reviews/editorials were not included, as were 2 other studies where there was no intervention and one where the timings were unclear. In further two papers, not all patients had ruptured ACLs. The author of one abstract [34] which met the inclusion criteria was contacted to request further information without success. 270 early procedures (< 3 weeks) were compared to 306 delayed procedures (≥ 3 weeks). The mean age in the early group was 29.0 ± 2.6 years, and 28.4 ± 2.3 years in the delayed group. A summary of included studies with outcome measures and timing is available in “Appendix 2” (Table 1).

**Table 1:** Demographics and study characteristics

Author and sample size	Injury to surgery interval		Age (years)		Follow-up	Graft	Follow-up (months)
	Early	Delayed	Early	Delayed			
1 Raviraj [26] (*n* = 105)	< 2 weeks	4–6 weeks	31.6 ± 5.3	31.2 ± 5.3	94 (*n* = 99)	STG	32
2 Li [27] (*n* = 38)	< 3 weeks	≥ 3 weeks	24.3 ± 4.9	26.5 ± 5.7	100 (*n* = 38)	ST	24
3^a^ Herbst [6] (*n* = 100)	1.1 ± 0.7 days	53.9 (SD ± 68.4 days)	27.6 ± 11.0	27.8 ± 10.6	99 (*n* = 99)	HT	24
3^b^ Herbst [6] (*n* = 60)	0.8 ± 0.8 days	49.2 (SD ± 86.3 days)	24.9 ± 7.9	24.7 ± 10.6	100 (*n* = 60)	HT	24
4 Manandhar [28] (*n* = 110)	< 3 weeks	42 (42–60 days)	n/s	n/s	96 (*n* = 106)	STG	6
5 Hur [29] (*n* = 91)	< 3 weeks	≥ 3 months	30.1	30.0	100 (*n* = 91)	HT	24
6 Karuppiah [30] (*n* = 87)	< 2 weeks	≥ 3 months	27.3 (15–48)	25.4 (15–46)	98 (*n* = 85)	NS	11

SD, standard deviation, graft type; STG, semitendinosus and gracilis; ST, semitendinosus; HT, hamstring tendon; NS, not stated

^a^Isolated ACL tear, ^b^combined ACL and meniscal tear

### Meta-analysis

A statistically significant difference of + 0.39 points on the Tegner activity scale was found in favour of earlier surgery. In this calculation the two arms of the Herbst 2017 [6] study (isolated ACL reconstruction^ and combined ACL reconstruction plus meniscal repair*) were analysed separately to permit a greater sensitivity in analysis. No differences were found between the groups for the patient-reported Lysholm scores. There was no statistically significant difference between intra-operative findings of meniscal lesions. A trend towards significance was observed for the incidence of chondral lesions in the early surgery group (Table 2).

**Table 2:** Results of meta-analyses of early versus delayed ACL reconstruction

Outcome	Papers	Relative risk (95% CI)	Overall effect (*p* value)	Heterogeneity (*I*^2^)
Tegner activity scale	2, 3^a^, 3^b^, 4, 5	0.39 (0.10, 0.67)*	0.008	0
Lysholm score	2, 3, 5	− 0.18 (− 2.40, 2.05)*	0.88	21
Meniscal lesion incidence	1, 2, 3^b^, 4, 5	0.84 (0.66, 1.08)	0.17	49
Chondral lesion incidence	1, 2, 4, 5	0.56 (0.31, 1.02)	0.06	73

*Mean difference (95 confidence intervals)

### PROMs

Four studies, all with similar designs, methodology and surgical technique [6, 26, 27, 29], assessed Tegner activity scale at the end of the follow-up period (26–36 months), reporting no differences between the groups on individual assessments. One further study [28] recorded Tegner activity scale at a much earlier final follow-up of 6 months with a trend approaching significance in favour of early 4.15 ± 1.45 versus delayed surgery 3.72 ± 1.34 (*p *= 0.06). Similarly, the four studies also assessed Lysholm scores at the same (26–36 months) follow-up with no differences found between the groups when assessed individually. One paper considered the International Knee Documentation Committee subjective rating score (IKDC) [35] with no difference between the groups, but with a trend towards significance in favour of early surgery when followed-up at 6 months (*p *= 0.08) [28]. Visual analogue pain scores (VAS) were reported in one study [6] with no differences found between the groups at any of the follow-ups up until the final reviews at 24 months.

### Objective

There was significant heterogeneity in both the follow-up times and the reporting methods for the recovery in range of motion (ROM). No significant differences were found between the groups when considering the mean time taken (14 weeks) to recover full ROM [26] or when ROM between groups was assessed at 6 and 24 months [27–29]. The only significant difference was found in one study at the midterm (12 months) follow-up in patients having combined ACL reconstruction and meniscal repair [6], where more patients in the delayed group had a lack of IKDC extension grade B (*p *= 0.04). Notably, however, this lack of extension was not present at either the 6 or 24-month follow-up in the same group, or at 6, 12 or 24 months in the parallel arm when patients underwent isolated ACL reconstruction.

Clinical examination by Lachman, pivot shift, and anterior draw testing showed no differences between the groups at any point up to the completion of follow-up [6, 26, 29]. On KT-1000 arthrometric evaluation of laxity at 24 months Li et al. [27] found evidence of greater stability with early surgery, but this was inconsistent with Raviraj et al. [26], who found no differences between the groups at a mean 32-month follow-up. Meniscal repair failures rates were assessed in 2 studies with divergent findings; in Karuppiah et al. [30] 23.0% failed after delayed and 4.8% following early surgery (*p *= 0.048), whilst Herbst et al. [6] found no differences in failure rates between the two groups. Objective IKDC [35] was not different between groups in two studies at final 24-month follow-up [6, 27].

### Critical appraisal

**Fig. 2:**
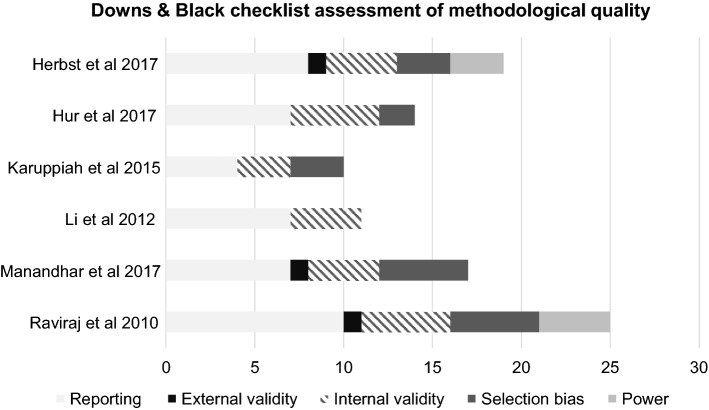
Summary of methodological characteristics of the included studies as per Downs and Black checklist [32]

Appraisal findings using the Downs and Black checklist [32] is graphically represented in Fig. 2. Two studies attempted randomisation; one via a computer-generated sequence [26], the second on basis of odd/even hospital numbers [28]. Whilst two studies attempted uniformity of follow-up using a single observer [26, 27], only one study was blinded to the timing of surgery [26]. Power calculations were performed in two studies, one a priori [26] and one post hoc [6]. Given the relatively low number of studies in any of the analyses, an assessment of publication bias via a funnel plot was considered to yield little value and was thus not performed.

## Discussion

This systematic review and meta-analysis reviewed new literature following a similar study in 2010 [16]. The study aimed to determine whether clinical outcomes up to 24 months post-ACL reconstruction were affected by the timing of surgery given updated surgical reconstruction methods and advances in rehabilitation protocols since 2010. In the meta-analysis, the only statistically significant finding was for the Tegner activity scale, which demonstrated improved reported outcomes with early surgery (*p *= 0.008). No other findings reached significance, which was in keeping with the previous 2010 Smith meta-analysis [16], and echoed in a subsequent systematic review of the timing for ACL reconstruction [17]. A further important finding was the numerous methodological limitations of the 6 studies included in this review, for example: incomplete or poor randomisation; limited follow-up of only 2 years; inadequate blinding; one study with a power calculation a priori; and an inability to accurately comment on publication bias. Accordingly, the results of this study, whilst based on all available evidence, should be interpreted with caution.

### PROMs

When assessed individually, there were no differences found in Tegner activity scales between the groups in any of the five papers. Mananadhar et al. [28] observed a trend towards significance in favour of early surgery at only the 6-month follow-up, whilst all the other similar studies reported no differences in outcomes from 12 to 24 months [26, 27, 29, 36]. Our meta-analysis found a 0.39 point greater Tegner activity scale score in the early surgical group (*p *= 0.008) compared with the delayed group, suggesting a potentially beneficial role for early surgery in the young active cohort under review. This finding contrasts with Smith’s 2010 meta-analysis [16] where no difference was recorded between the groups; however, the cohorts in that analysis had a maximum of 70 patients and none of the studies were adequately powered. With no objective value for what constitutes a minimum clinically important difference (MCID) in Tegner activity scale [37, 38], the observed effect size of 0.39 must be taken in context. In a review of measures of knee function, Collins 2011 [37] reported that post-ACL reconstruction score differences of 0.74 (were classed as moderate) and 1.0 (as large differences) at 6 and 12 months, respectively; thus, the observed effect of 0.39 is likely to equate to a negligible or small clinical difference and should not be the basis for any change in clinical practice.

Four papers reported Lysholm scores and found no differences between the groups when assessed separately or in the meta-analysis. As a validated and reliable instrument [39] and the most widely used subjective assessment of knee function worldwide [40], the score is a key tool in the evaluation of outcomes within and between studies. With only short-term (2-year) follow-up available, a cautious interpretation is mandated, and medium (5-year) and long-term (10-year) [41] data might help to better inform decisions on the timing of ACL reconstructions.

In papers reviewing objective [6, 27], and subjective [28] IDKC, no differences were found between the groups. In a systematic review, Wera 2014 [40] evaluated the use of both IKDC forms and recommended its interpretation in combination with the Tegner activity scale [40]. In our study, the IKDC findings reflected the Tegner scores, and no differences were found between the groups. One author assessing pain scores with VAS found no differences [6]. The VAS is a unidimensional measure with excellent test–retest reliability for chronic painful musculoskeletal conditions [42], but may be less valid in ligamentous knee injuries [43, 44].

### ROM and stability

Five papers assessed post-operative ROM, but due to considerable heterogeneity in measurement methods, a meta-analysis was not indicated and a narrative review was preferred. In the 1990s, rehabilitation protocols typically focused on restricting ROM and surgical intervention utilised non-anatomical ACL reconstruction—factors which may account for reduced ROM noted in studies during this period [46, 47]. Through Shelbourne’s 1990 findings that patients who were non-compliant with their rehabilitation programme and who ambulated on their reconstructed knee without splints had fewer ROM problems with better strength gains [45], various accelerated rehabilitation programmes were developed and became the norm after support in multiple subsequent systematic reviews [46–49]. The five papers in this study measuring post-operative ROM utilised an accelerated rehabilitation protocol which may explain why no differences were found between the groups at final follow-up in any study (ranging 14 weeks to 24 months); this is in keeping with findings from similar studies with accelerated protocols [36, 50–53].

Two studies objectively assessed stability scores on a KT-1000 arthrometer with contrasting results. In the smaller study [27] which failed to score for external validity, selection bias or power on the checklist, the authors reported significantly greater laxity in the delayed surgery group. However, the authors of the paper with the highest methodological score in this review conducted randomisation, assessor blinding, and an adequate power calculation, and actually reported no differences between the groups (*p *= 0.9) [26]. This is in keeping with the wider literature where no difference was evident in a meta-analysis [16], suggesting the findings of Li et al. [27] should be interpreted in context of methodological compromise.

### Chondral and meniscal lesions

Proponents of early reconstruction cite the odds of a knee cartilage lesion increasing by almost 1% per month in ACL-deficient knees between time of injury and surgery [19], as a driver to early surgery; cartilage lesions and untreated meniscal tears are recognised predictors for OA [20, 21, 54]. When meta-analysing the incidence of chondral lesions, there was a borderline significant (*p *= 0.06) effect favouring early surgery, whilst there was no apparent effect on the incidence of meniscal lesions.

Our definition of delayed surgery might be considered ‘early’ by some [24, 55–60]. The longest delay from injury to surgery in all studies in this review was 74 weeks [29], and subsequently, the early versus delayed heterogeneity may be too small to permit any significant findings. This is reflected in the literature where four authors with similar definitions of early (< 3 weeks) and delayed (≥ 3 weeks) interventions failed to find any differences between groups [26, 36, 50, 53]. These results are contrasted in papers utilising a later definition of ‘early’ to include periods up to 5 months post-injury, and ‘delayed’ ranging from 3 months to 14 years. Across studies utilising these later/longer timeframes all five authors reported a significantly higher incidence of meniscal/chondral defects in the delayed groups. Thus, whilst no statistically significant findings are present between the groups in our review, on the balance of current evidence, a difference might have existed if surgery had been delayed by months or years.

Although lesions noted in this study may remain clinically silent for decades in many patients, their presence is a cause for concern given the increased risk of OA with its associated physical, psychological and financial impact [61–63]. In a methodologically robust study, Frobell et al. [24] found no differences between early ACL reconstruction and non-surgical treatment with the option for a later reconstruction, initially indicating that a ‘watch and wait’ approach might be permissible. However, there were markedly increasing proportions of reconstructions in the delayed groups of 7% at 6 months, 20% at 12 months and 51% at midterm (5-year) follow-up, with more frequent meniscal signs, symptoms and knee instability in the ‘optional delayed’ reconstruction group (*p *< 0.001) [25]. Thus, whilst there were no differences in observed meniscal/chondral lesions between early or delayed surgery groups seen in our review, on the basis of the wider current literature, early intervention would appear to confer a decreased risk of developing later meniscal/chondral lesions and potentially might then lower the subsequent risk of OA.

### Meniscal repair failure rate

Assessment of meniscal repair failures rates yielded contrasting findings in two papers. Karuppiah et al. [30] retrospectively analysed the notes of patients undergoing combined ACL reconstruction plus meniscal repair and noted a higher failure rate in delayed versus early repairs (*p *< 0.05). Herbst et al. [6] found no difference between early (mean < 1 day) and delayed (mean 49.2 days) surgery. The size and locations of the tears was only reported in the retrospective analysis, thus a direct comparison is difficult. One should bear in mind that the discrepancy in tear rates is likely to relate to the small study sizes (of approximately 100 patients), which were not adequately powered to detect a difference. Additionally, there is no single accepted definition of what constitutes a failure in meniscal repair, how one identifies when those patients represent and how one actually makes a correct diagnosis.  In addition, a maximum of 2-years follow-up is probably too short a time to make a true judgement. Given that the inclusion/exclusion criteria, patient demographics, surgical technique and instrumentation are actually similar, and with the recognised increased risks of OA with meniscal lesions, an urgent well-designed randomised control trial is required to determine whether timing of intervention actually affects the rates of meniscal repair failure.

### Limitations

There are substantial limitations to this paper. A solely electronic search of three databases (Medline, Embase, OpenGrey) was performed. The reviewing author was not blinded to the journal titles, the authors of the studies, or sources. Arbitrary time frames of early and delayed reconstructions were applied based on previous meta-analysis, with a minor modification. The degree of ACL tear and presence or absence of meniscal lesions were inconsistently reported across the studies with no controls applied. The follow-up periods were limited to a maximum of 2 years.

## Conclusion

The primary finding from our meta-analysis was that based on the literature published since the last systematic review [16] there was a statistically significant, though small, difference in Tegner activity score in favour of early over delayed ACL reconstruction. Whether the magnitude of this improvement is sufficient to translate into any clinically meaningful difference, or influence surgical practice, is unclear.

No differences were observed in Lysholm scores or the incidence of meniscal/chondral lesions between the groups. A secondary finding was the low external validity, the risk of selection, performance and detection bias, and the potential for type II statistical errors across the reviewed studies, with only one series performing satisfactory sample size calculations [26]. Given the sub-optimal methodological quality of the included studies, one must exercise some caution when interpreting the conclusions of this review. Finally, this paper was only able to review the outcomes up to 2 years, and an adequate long-term follow-up of 10+ years is required in order to better appreciate the effects of delaying ACL reconstruction and to build on the work of Frobell et al. [24, 25]. To attempt a definitive answer, either a methodologically robust randomised control trial or a concerted effort to unlock the potential of the UK National Ligament Registry (NLR) is required.

## Appendix 1: Search terms for Medline, Embase and OpenGrey

### Medline (Ovid)

1. ANTERIOR CRUCIATE LIGAMENT/su
2. ANTERIOR CRUCIATE LIGAMENT RECONSTRUCTION/
3. (“anterior cruciate ligament*” or ACL*).ab,ti.
4. (reconstruct* or repair*).ab,ti.
5. 3 and 4
6. 1 or 2 or 5
7. TIME FACTORS/
8. (time or timing or early or late or delay* or week* or day or days).ti.
9. 7 or 8
10. 6 and 9
11. limit 10 to yr = ”2009 Current”

### Embase

1. ANTERIOR CRUCIATE LIGAMENT/su
2. ANTERIOR CRUCIATE LIGAMENT RECONSTRUCTION/
3. (“anterior cruciate ligament*” or ACL*).ab,ti.
4. (reconstruct* or repair*).ab,ti. 
5. 3 and 4
6. 1 or 2 or 5
7. TIME FACTORS/
8. (time or timing or early or late or delay* or week* or day or days).ti.
9. TIME TO TREATMENT/
10. 7 or 8 or 9
11. 6 and 10
12. limit 11 to yr = ”2009 -Current”

### OpenGrey

1. Anterior cruciate ligament reconstruction

## Appendix 2: Studies and time frames of outcome measures

**Table:**

Authors	Design	*Primary outcome* Secondary outcome(s) (all timings post-operative unless otherwise stated)
1. Raviraj et al. [26]	Randomised control trial	*ROM*—*4, 8, 12 and 14* *weeks* Lysholm score—at 26–36 months Tegner score—at 26–36 months Stability—Lachman, pivot shift, anterior draw, varus/valgus test, KT-1000 arthrometer at 26–36 months Meniscal injury—at time of surgery Chondral injury—at time of surgery
2. Li et al. [27]	Retrospective cohort	*Lysholm score*—*preoperative and 24* *months* Tegner score—preoperative and 24 months IKDC score—preoperative and 24 months ROM—preoperative and 24 months Stability—kneelax arthrometer—preoperative and 24 months Meniscal/chondral lesion—at time of surgery
3^. Herbst et al. [6] isolated ACL lesion	Prospective non-randomised trial	*Lysholm score*—*preoperative and 24* *months* IKDC score—preoperative and 24 months VAS pain—preoperative and 24 months ROM—preoperative and 24 months Stability—Lachman, anterior draw, pivot shift—preoperative and 24 months Cyclops lesion—at time of surgery Meniscal repair failure—within 24 months ACL graft rupture—within 24 months IKDC objective—at 24 months
3*. Herbst et al. [6] combined ACL and meniscal lesion	Prospective non-randomised trial	*Lysholm score and Tegner*—*preoperative and 24* *months* IKDC score—preoperative and 24 months VAS pain—preoperative and 24 months ROM—preoperative and 24 months Stability—Lachman, anterior draw, pivot shift—preoperative and 24 months Meniscal injury—at time of surgery Cyclops lesion—at time of surgery Meniscal repair failure—within 24 months ACL graft rupture—within 24 months IKDC objective—at 24 months
4. Manandhar et al. [28]	Randomised control trial	*ROM*—*3, 6, 12 and 24* *weeks* Tegner—6 months IKDC subjective—6 months Meniscal/chondral lesion at time of surgery
5. Hur et al. [29]	Prospective non-randomised trial	*Meniscal/chondral lesions at time of surgery* ROM—at 24 months Tegner—pre-injury and 24 months Meniscal/chondral lesion—at time of surgery Muscle power—at 24 months Pivot shift—preoperative and 24 months Lachman—preoperative and 24 months IKDC—variant not stated
6. Karuppiah et al. [30]	Retrospective cohort	*Meniscal repair failure*—*at 11* *months* Post-operative complications—at 11 months

## Appendix 3: Meta-analysis output for four outcomes measures

### Meta-analysis of Tegner activity scale mean difference early versus delayed

**Table:**

Study or subgroup	Early			Delayed			Weight (%)	Mean difference IV, fixed, 95% CI	Mean difference IV, Fixed, 95% CI
	Mean	SD	Total	Mean	SD	Total			
Herbst et al. [6]	6.7	1.3	50	6.3	1.4	50	28.9	0.40 [− 0.13, 0.93]	
Herbst et al. [6]	6.6	1.2	30	6.3	1.5	30	17.2	0.30 [− 0.39, 0.99]	
Hur et al. [29]	6	1.6	48	5.6	1.5	43	20.0	0.40 [− 0.24, 1.04]	
Li et al. [27]	6.6	1.9	17	6.3	1.8	21	5.8	0.30 [− 0.89, 1.49]	
Manandhar et al. [28]	4.15	1.45	53	3.72	1.34	51	28.2	0.43 [− 0.11, 0.97]	
Total (95% CI)			198			195	100.0	0.39 [0.10, 0.67]	
Heterogeneity. *χ*^2^ = 0.11, *df *= 4 (*p* = 1.00); *I*^2^ = 0%									
Test for overall effect: Z = 2.65 (*p* = 0.008)									

### Meta-analysis of Lysholm score mean difference early vs delayed

**Table:**

Study or subgroup	Early			Delayed			Weight (%)	Mean difference IV, Random, 95% CI	Mean difference IV, Random, 95% CI
	Mean	SD	Total	Mean	SD	Total			
Herbst et al. [6]	93.1	8.5	50	92.4	6.8	50	39.9	0.70 [− 2.23, 3.72]	
Hur et al. [29]	94.5	8.9	48	96.3	3.7	4.	45.5	− 1.80 [− 4.55, 0.95]	
Li et al. [27]	94.7	9.3	17	92.2	7.8	21	14.6	2.50 [− 3.04, 8.04]	
Total (95% CI)			115			114	100.0	− 0.18 [− 2.40, 2.05]	
Heterogeneity. *τ*^2^ = 0.98; *χ*^2^ = 2.55, *df *= 2 (*p* = 0.28); *I*^2^ = 21%									
Test for overall effect: *Z* = 15 (*p* = 0.88)									

### Risk of meniscal lesions early versus delayed

**Table:**

Study or subgroup	Early		Delayed		Weight (%)	Risk ratio	Risk ratio
	Events	Total	Events	Total		M–H, random, 95% CI	M–H, random, 95% CI
Herbst et al. [6]	30	80	30	80	20.4	1.00 [0.67, 1.49]	
Hur et al. [29]	25	48	27	43	23.0	0.83 [0.58, 1.18]	
Li et al. [27]	2	17	9	21	2.9	0.27 [0.07, 1.10]	
Manandhar et al. [28]	22	51	34	51	22.1	0.65 [0.45, 0.94]	
Raviraj et al. [26]	38	51	35	48	31.6	1.02 [0.81, 1.29]	
Total (95% CI)		247		243	100.0	0.84 [0.66, 1.08]	
Total events	117		135				
Heterogeneity. *τ*^2^ = 0.04; *χ*^2^ = 7.79, *df *= 4 (*p* = 0.10); *I*^2^ = 49%							
Test for overall effect: *Z* = 1.36 (*p* = 0.17)							

### Risk of chondral lesions early versus delayed

**Table:**

Study or subgroup	Early		Delayed		Weight (%)	Risk ratio	Risk ratio
	Events	Total	Events	Total		M–H, random, 95% CI	M–H, random, 95% CI
Hur et al. [29]	15	48	20	43	30.9	0.67 [0.4., 1.14]	
Li et al. [27]	0	17	7	21	4.1	0.08 [0.00, 1.33]	
Manandhar et al. [28]	10	53	28	51	28.6	0.34 [0.19, 0.63]	
Raviraj et al. [26]	29	51	31	48	36.4	0.88 [0.64, 1.21]	
Total (95 CI)		169		163	100.0	0.56 [0.31, 1.02]	
Total events	54		86				
Heterogeneity. *τ*^2^ = 0.23; *χ*^2^ = 11.05, *df *= 3 (*p* = 0.01); *I*^2^ = 73							
Test for overall effect: *Z* = 1.89 (*p* = 0.06)							
